# How Do Male Football Players Meet Dietary Recommendations? A Systematic Literature Review

**DOI:** 10.3390/ijerph19159561

**Published:** 2022-08-03

**Authors:** Karol Danielik, Anna Książek, Aleksandra Zagrodna, Małgorzata Słowińska-Lisowska

**Affiliations:** Department of Biological and Medical Basis of Sport, Faculty of Physical Education and Sports, Wroclaw University of Health and Sport Sciences, 51612 Wrocław, Poland; karoldanielik@gmail.com (K.D.); aleksandra.zagrodna@awf.wroc.pl (A.Z.); malgorzata.slowinska-lisowska@awf.wroc.pl (M.S.-L.)

**Keywords:** dietary intake, soccer players, nutritional recommendations, sports nutrition

## Abstract

The aim of this review was to determine whether male football players meet dietary recommendations according to a UEFA expert group statement and to identify priority areas for dietetic intervention, including training periodization and field position. A database search of PubMed, Web of Science, EBSCO and Scopus was performed. To be included within the final review, articles were required to provide a dietary intake assessment of professional and semi-professional football players. A total of 17 studies met the full eligibility criteria. Several studies showed insufficient energy and carbohydrate intake compared to the recommendations. A majority of athletes consume adequate protein and fat intakes compared to the recommendations. In addition, several studies showed the insufficient intake of vitamins and minerals. This systematic review showed that football players do not meet the nutritional recommendations according to the UEFA expert group statement. Future research should be focused on how to apply nutritional recommendations specific for athletes in accordance with training periodization and positions on the field.

## 1. Introduction

Football is a sport discipline characterized by high-intensity efforts followed by periods of active recovery or passive rest. Football players complete a weekly microcycle of 8–10 training units during the preparation period. The competitive period is characterized by the highest-intensity efforts, which increase energy expenditure [1]. The match effort, on the other hand, is characterized by a variable tempo, during which the athlete jogs, sprints and dynamically changes the direction of running, performing about 726 different actions of movements and turns [2,3,4]. The number of matches in a season at an elite level number up to 60 [5], and players run about 9–12 km in each of them [6]. This distance depends mainly on the players’ position on the field [3,7]. It is also worth noting that energy expenditure during a match is dependent on the position of the player [8] and that energy and nutrient requirements need to be individualized [9].

Properly balanced nutrition is one of the factors that support performance enhancement and post-exercise recovery [10,11]. An adequate nutrient supply contributes to training adaptation [12,13] and can have an impact on reducing the risk of injury and recovery time [14]. Training periods vary in terms of physiological demands and differences in energy expenditure generated. Planning the nutrition of athletes with adjustments to the periods of training and individualization requires special attention [15]. Improvement in an athlete’s exercise capacity is influenced not only by the appropriate choice of exercise load but also by the use of appropriate nutritional strategies to maximize adaptive changes [16,17]. 

Recently, it has been observed that physiological demands in football have increased [2,18]. Evidence suggests that high-intensity and sprint activities have increased by 30–80% [2]. Hence, nutritional strategies are rated by elite teams as one of the most important ways to accelerate this process. In 2017, Collins et al. [19] indicated that specific nutrition guidelines for football had not been updated for over a decade [20]. Three years later, an UEFA expert group statement on nutrition in elite football has been published, highlighting that increased interest in and focus on nutrition brings with it greater challenges in football society [21]. The authors indicated that this nutritional guideline represented a vital first step, bringing together the best scientific and on-field practitioners from around the world to add much-needed continuity to recommendations for players and teams. The continued development of good nutritional practices within the game will also have an impact far beyond clubs’ training grounds, helping to promote good health across society as a whole [21].

Therefore, the aim of this review was to determine whether male football players meet the dietary recommendations according to the UEFA expert group statement, and to identify priority areas for dietetic intervention, including training periodization and field position.

## 2. Materials and Methods

The review is reported using the preferred reporting items for systematic reviews and meta-analyses (PRISMA) guidelines [22]. Population, intervention, comparison, outcome and study design (PICOS) criteria are defined in Table 1. All keywords used in the literature search are listed in Table 2. Due to the updated nutritional recommendations for football players, the data from all studies were compared to the UEFA expert statement [21] in the discussion section.

### 2.1. Search Strategy

One author (KD) searched PubMed (MEDLINE), Web of Science, Scopus and EBSCO (including SPORTDiscus) databases for literature relevant to the aims of the systematic review.

### 2.2. Eligibility Criteria

All original studies (including cross-sectional studies, longitudinal studies, observational studies and randomized controlled trials) were included in the study. Studies not written in English were excluded from the analysis. Studies published prior to 2011 were excluded from the analysis. Only studies with adult male professional or semi-professional footballers as participants were eligible for the review. “Professional” athletes are defined as individuals who exercise > 10 h/week and whose athletic performance has achieved the highest level of competition, and “semi-professional” athletes exercise > 6 h/week with an emphasis on improving performance. Only studies reporting typical consumption values (e.g., energy as kcal/day, protein as g/day or calcium as mg/day) were included. Studies that qualitatively assessed diet were not included in the review. All inclusion and exclusion criteria are specified in Table 3.

### 2.3. Study Selection Process

**Figure 1:**
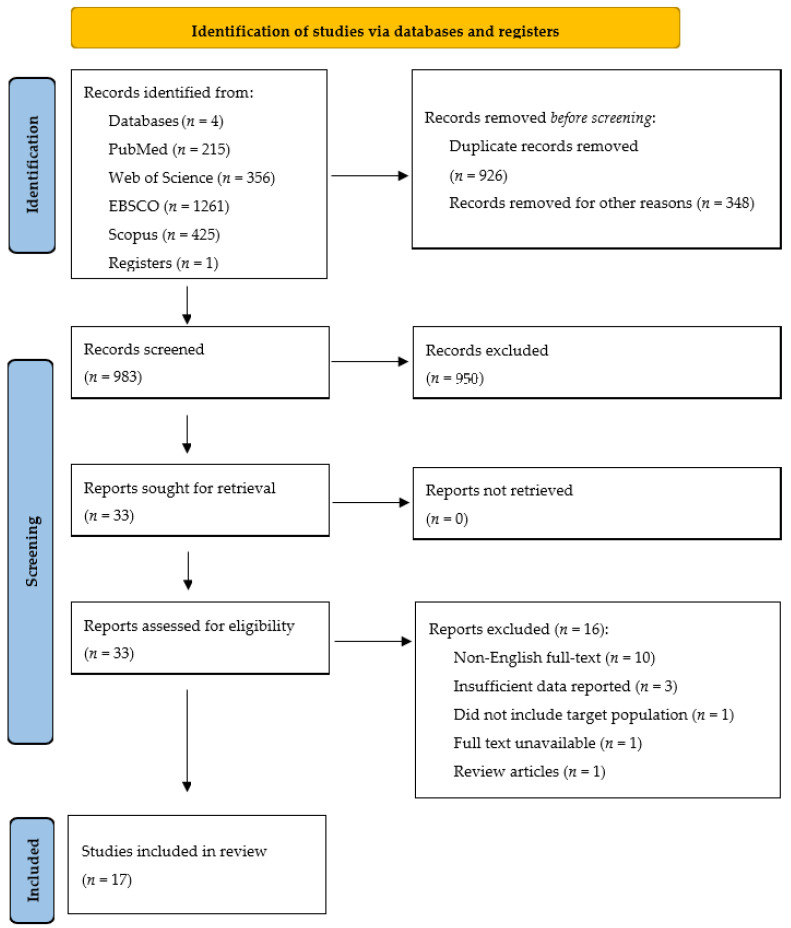
Study selection process.

Publications were initially reviewed based on the title and abstract by two authors (K.D. and A.K.). Duplicates and articles deviating from the topic of the literature review were removed. All articles that passed title and abstract review were subjected to full-text review. Each full-text article was checked against the inclusion and exclusion criteria presented in Table 3 by two authors (K.D. and A.K.). Differences of opinion regarding the inclusion of articles in the literature review were verified by the third author (A.Z.). The stages of article selection and reasons for excluding papers are shown in Figure 1.

### 2.4. Study Quality: Risk of Bias

The quality of individual studies included in the review was assessed for bias (quality) using the Academy of Nutrition and Dietetics Evidence Analysis Manual [23], which permits the assessment of relevance and validity, with the allocation of either a positive, neutral or negative quality ranking. All studies were compared against this checklist by two different reviewers (K.D., A.K.). Only studies that accurately described study selection, including inclusion/exclusion criteria, were included in the review. At end stage, a third reviewer (A.Z.) reviewed any discrepancies. In quality assessment, no studies had negative rating; therefore, all of the obtained articles were included.

## 3. Results

### 3.1. Study Selection

The search retrieved 2257 studies for review. After duplicates were removed, 1274 studies were retained. Thirty-three studies were retrieved and their titles and abstracts screened. Seventeen [24,25,26,27,28,29,30,31,32,33,34,35,36,37,38,39,40] of these met the inclusion criteria and were included in the review. Reasons for study exclusions are highlighted in Figure 1. No additional studies were identified through the hand-searching of reference lists or manual searches using Google Scholar.

### 3.2. Study Characteristics

The characteristics of the athletes from the papers included in the systematic literature review are presented in Table 4. Study participants were from England [25,37,38,39], Australia [31,32,36], the Netherlands [34,35], Malta [33], Spain [30,40], Poland [29], Korea [27,28], Japan [26] and Brazil [24]. The majority of studies included in this review were observational in design and sometimes cross-sectional (*n* = 14), with the remaining being case studies [39], case control [26] or longitudinal [32]. In eight papers, dietary intake during the preparation period [24,26,27,28,29,31,32,40] was evaluated, and 10 papers concentrated on the starting period [25,30,32,33,34,35,36,37,38,39]. Only one study analyzed nutrient intake in both training periods (pre-season and in-season) [32]. Only papers that assessed nutrient intake using validated methods that quantify intake (food diaries, 24-hour dietary recall, weighted dietary records, semi-structured food records) were included in the systematic review. Most of the papers evaluated dietary intake over a period of 7 days [26,27,28,29,37,38,39,40] and 3 days [24,31,32,33,34,36]. Two papers evaluating nutrient intake in athletes for 4 days [25,35] and 1 paper assessing intake for 6 days [30] were also taken into consideration.

### 3.3. Energy Value

The average energy and macronutrient intake analyzed in the papers are shown in Table 5 and Table 6. 

The mean energy content in the diet in all analyzed studies was 2813 ± 446 kcal/day (37.7 ± 6.2 kcal/kg BM/day), with the lowest being 2164 ± 498 kcal/day (28.6 ± 6.6 kcal/kg BM/day) [33] and the highest being 3789 ± 532 kcal/day (47.7 ± 6.6 kcal/kg BM/day) [37]. Several studies provided evidence that the energy intake of football players [30,31,32,33,34,36,41] did not meet the recommended energy requirements advocated by the UEFA expert group statement (2900–3500 kcal/day) [21].

Energy intake during the pre-season was assessed in 8 studies [24,26,27,28,29,31,32,40], with a mean intake of 2916 ± 471 kcal/day (40.5 ± 7.6 kcal/kg BM/day) kcal. The lowest energy intake was 2246 ± 549 kcal/day (29.7 ± 7.3 kcal/kg BM/day) [32], and the highest was 3456 ± 435 kcal/day (49.6 ± 6.2 kcal/kg BM/day) [28].

A total of 10 studies evaluated energy intake during the competitive period [25,30,31,33,34,35,36,37,38,39], in which the average intake was 2758 ± 422 kcal/day (36.0 ± 4.4 kcal/kg BW/day). The lowest intake was 2164 ± 498 kcal/day (28.6 ± 6.6 kcal/kg BW/day) [33], and the highest was 3789 ± 532 kcal/day (47.7 ± 6.6 kcal/kg BW/day) [37].

Table 7 and Table 8 present energy and nutrient intake by position on the field for two periods (pre-season, in-season) [24,30,34,37,39,40]. In the pre-season, the lowest energy intake was recorded in midfielders 2534 ± 550 kcal/day (35.3 ± 7.7 kcal/kg BW/day) [34], and the highest in central midfielders 3701 kcal/day (47.2 kcal/kg BW/day) [37]. It was also observed that energy intake in goalkeepers was below 30 kcal/kg BW/day in the pre-season (2914 kcal/day, 19.2 ± 7.5 kcal/kg BW/day) [40] and in-season (2606 ± 586 kcal/day, 29.1 ± 6.5 kcal/kg BW/day) periods [34].

### 3.4. Macronutrients

#### 3.4.1. Carbohydrates

The mean total CHO-intake content in the diet of all analyzed studies was 327 ± 94 g/day (4.2 ± 1.2 g/kg BM/day); the lowest total CHO intake was 213 ± 107 g/day (2.8 ± 1.4 g/kg BM/day) [32], and the highest total CHO intake was 508 ± 152 g/day; 4.2 ± 1.4 g/kg BM/day [37]. Out of the 15 studies that provided mean intake of CHO, 6 demonstrated [31,32,33,34,36,39] low CHO-intake and fell below the standards of the UEFA expert group statement (4–8 g/kg BM/day) [21].

Total CHO intake during the preparation period was assessed in six studies [24,26,29,31,32,40] and was equal to 340 ± 92 g/day (4.7 ± 1.4 kcal/kg BM/day) (range 210 ± 76–452 ± 162 g/d; 2.9 ± 1.1–6.9 ± 2.4 g/kg BM/day). On the other hand, it was 326 ± 93 g/day (range 213 ± 107 g/day; 2.8 ± 1.4 g/kg BM/day [32]—508 ± 152 g/day; 4.2 ± 1.4 g/kg BM/day [37]) during the competitive period [25,29,30,32,33,34,35,36,37,38,39]. In the preparatory period, inadequate CHO intake was found in two studies [31,32] compared to the recommendations (4–8 g/kg BM/daily) [21]. Seven studies conducted in the competitive period [30,32,33,34,35,36,39] reported inadequate CHO intake that was below the recommendations (6–8 g/kg BM/daily) [21].

Two papers [24,40] concerning the pre-season and three papers [30,34,39] on the in-season assessed CHO intake in relation to on-field positions. The lowest average intake of CHO in the pre-season was shown in goalkeepers (320 ± 12 g/day) and the highest in defenders (419 ± 98 g/day) [40]. In season, the lowest intake was recorded in goalkeepers (222 ± 54 g/day; 2.6 ± 0.6 g/kg BM/day) [39] and the highest in side midfielders (352 ± 54 g/day, 4.9 ± 0.8 g/kg BM/day), central midfielders (352 ± 72 g/day; 4.9 ± 1.3 g/kg BM/day) and forwards 352 ± 54 g/day 4.9 ± 0.8 g/kg BM/day) [30].

#### 3.4.2. Protein

The average protein intake in the analyzed papers was 1.9 ± 0.3 g/kg BM/day (range 1.3 ± 0.4 g/kg BM/day [26]–2.5 g/kg BM/day [38]). Three studies [26,29,33] showed lower protein intake, and in one paper [39], protein intake exceeded the protein recommendations advocated by the UEFA expert group statement (1.6–2.2 g/kg BM/daily) [21]. The adequate intake of protein was observed in the other papers.

In the pre-season period, protein intake was assessed in six papers [24,26,29,31,32,40]. The average protein intake during the preparation period was 1.7 ± 0.3 g/kg BM/day ranging from 1.3 ± 0.4 g/kg BM/day [26] to 2.0 ± 0.7 g/kg BM/day [40]. In the in-season period, protein intake was assessed in eight studies [30,32,33,34,35,36,38,39]. The average protein intake during the competitive period was 2.0 ± 0.3 g/kg BM/day (range 1.5 ± 0.4 g/kg BM/day [33]—2.5 g/kg BM/day [38].

In the pre-season period, the highest protein intake was shown in central midfielders (164 g/day; 2.3 ± 1.0 g/kg BM/day) and the lowest in forwards (101 ± 16 g/day; 1.4 ± 0.2 g/kg BM/day) [24]. A study by Iglesias-Gutiérrez et al. showed the lowest protein intake (115 ± 29 g/day; 1.5 ± 0.4 g/kg BM/day) in goalkeepers [30]. In contrast, Anderson et al. reported the highest protein intake (207 ± 36 g/day; 2.4 ± 0.4 g/kg BM/day) in goalkeepers [39].

#### 3.4.3. Fats

Fat intake was assessed in 11 studies [24,26,29,30,31,32,33,34,35,36,40]. The mean proportion of dietary fat in the analyzed papers was 31.1 ± 3.0% (range 25 ± 3% [29]–37 ± 5% [30]). One study [30] showed that fat intake by football players exceeded the recommendations advocated by the UEFA expert group statement (20–35% of total dietary energy) [21].

The average proportion of fats in the total daily ratio during the preparation period was 30.5 ± 4.0% and during the in-season period 31 ± 2%. The proportion of energy from fat in the diet in the pre-season period ranged from 25 ± 3% [29] to 34 ± 12% [32]. During the in-season period, the proportion of energy from fat in the papers analyzed was 29 ± 4% [35] to 37 ± 5% [30].

Five studies evaluated fat intake dependent on the position occupied on the field, of which two [24,40] concerned the preparation period and three the competitive period [30,34,39]. The lowest fat intake during the preparation period was recorded in center defenders—fullbacks—(1.0 ± 0.4 g/kg BM/day) [24] and the highest in midfielders (1.4 ± 0.6 g/kg BM/day) [24]. During the season, the lowest proportion of dietary fat was recorded in goalkeepers (0.9 ± 0.2 g/kg BM/day) [34]. In contrast, the Anderson et al. [39] study found that goalkeepers consumed the highest amount of fat (1.9 ± 0.4 g/kg BM/day).

### 3.5. Vitamins and Minerals

The average intake of vitamins and minerals in the diets of athletes was analyzed in four papers [24,26,29,40]. All studies included in the review evaluating vitamin and mineral intake were from the pre-season period [24,26,29,40]. In the analyzed studies, the insufficient intake of magnesium, calcium, zinc, folic acid and vitamins such as B_1_, B_2_, B_12_, A, C, E and D was compared to the recommended standards [42,43,44]. Książek et al. [29] analyzed the average intake of vitamins and minerals with respect to the supplementation used. It was shown that the mean intake of folic acid, vitamin D, vitamin E, iron and zinc in the diet that included supplementation was consistent with the standard intake.

Four studies assessing iron and calcium intake and three articles assessing vitamin D intake were included in the systematic review. The average reported daily intake of iron was 14.3 ± 3.8 mg/day. Two out of four studies [29,40] reported intake that met the iron intake requirement, which is 15–18 mg/day. The average daily calcium intake from studies was 1027 ± 323 mg/day. In three of the four papers, insufficient calcium intake was observed in relation to recommendations [24,26,29]. Three studies reported vitamin D intakes of 12.2 ± 8.7 µg/day [40], 3.1 µg/day [24] and 4.9 µg/day (56.5 µg/day after accounting for supplementation) [29]. With regard to the Institute of Medicine (US) Committee to Review Dietary Reference Intakes for Vitamin D and Calcium (15 µg/day, 1300 mg/day) [44], only 1 paper [29] reported the recommended intake.

## 4. Discussion

This review aimed to assess the adequacy of dietary intake in male professional and semi-professional football players when compared to the dietary recommendations advocated by UEFA expert group statement and to define dietary areas demanding improvement. This systematic review found that football players’ total energy and carbohydrate intake did not meet sports-nutrition recommendations; on the other hand, the majority of athletes had adequate protein and fat intake.

### 4.1. Energy Intake in Football Players

Adequate energy supply is a crucial factor for enhancing physiological adaptations during the pre-season and for optimizing performance in-season. According to the UEFA expert group statement [21], the mean daily energy expenditure of goalkeepers and outfield players has been estimated at ~2900 kcal/day and ~3500 kcal/day, respectively, with match-day energy expenditure estimated at ~3500 kcal. Several studies (*n* = 7) showed that football players’ energy intake does not meet the demands of the pre-season [29,31,32] or the in-season [30,32,33,34,36]. Inadequate energy supply in the diets of the athletes studied may have been due to inadequate reporting related to a lack of recording and undernutrition during the study [45]. Methods used to measure dietary intake are hampered due to errors in precision (repeatability, reproducibility and reliability) and validity (accuracy). Moreover, existing literature has shown significant variability between methods used to evaluate dietary intake, with the frequent underreporting and misreporting of consumption [46].

Another reason for insufficient energy supply may be a real problem associated with low energy availability (LEA). Inadequate energy supply in the diets of athletes may contribute to relative energy deficiency in sport (RED-S). RED-S is a common and contemporary topic in sports nutrition and is defined as a range of signs and symptoms that may negatively affect the health and performance of physically active individuals (both female and male). The effects include endocrine, physiological, metabolic and psychological dysregulation, which may ultimately affect physical capacity and performance [47]. There is little research concerning RED-S in football players, and this has so far focused only on female football players [48].

Excessive energy intake relative to the daily requirements may contribute to weight gain by increasing the proportion of body fat. Excess body fat negatively affects acceleration capacity, overall energy expenditure and injury risk. It is important that sports nutritionists and performance teams provide players with body-composition targets [21]. Such targets can be achieved using periodized nutrition. Periodizing nutrition focuses on the different energy needs and varying intake during different phases of the year [16]. In football, energy demands are lower in the in-season and higher in the pre-season [1], when the nutritional strategy should be focused also on obtaining optimal body composition [49].

### 4.2. Carbohydrate Intake in Football Players

Due to football involving activities of varying intensities, including walking, jogging, sprinting, changing direction, jumping, striking the ball and contact with opposition players, CHO are the primary fuel during matches [21].

During the pre-season, the main task of a football player is to improve their athletic performance, to avoid injury and illness and to prepare to play throughout the season. The maintenance of football players’ athletic performance largely depends on the availability of CHO as energy substrates [50]. The suggested CHO intake is 4–8 g/kg BM/day and depends on variations in loads and individual training goals (such as maintaining or changing body composition). If there were a greater intensity and volume of training, higher CHO intakes (6–8 g/kg BM/day) would likely be required. Only in two of the six analyzed papers [31,32] did not players consume CHO according to the recommendations (4–8 g/kg BM/day) during the pre-season.

In season, the maintenance of maximal performance in football players is highly related to the replenishment of glycogen stores via proper CHO intake. Currell et al. [51] showed that CHO intake during matches improved running performance (agility, dribbling and kicking accuracy). In-season CHO recommendations suggest 6–8 g/kg BM/day prior to a match and when there is fixture congestion (a 2–3-day period between games) [21]. One [37] of two papers evaluating match days showed an optimal CHO supply (6–8 g/kg BM/day).

The optimum physique varies according to a player’s physiology, their field position and playing style [21]. Although goalkeepers are usually taller and have higher body mass (higher fat mass) than field players [52], they still have the optimum physique, in terms of position-specific and seasonal trends. Moreover, athletes have different energy requirements depending on their position on the field [53]; therefore, the amount of CHO should be adjusted individually for each athlete. Players whose positions on the field are characterized by a higher energy expenditure should increase their daily intake of CHO.

### 4.3. Protein Intake in Football Players

According to the UEFA expert group statement [21], to enhance training adaptation, support recovery and stimulate muscle protein synthesis, the recommended protein intake is 1.6–2.2 g/kg BM/day. In this review, the majority of studies reported that protein intake matched the recommendations for the pre-season [24,31,32,40] and for the competitive period [30,32,34,35,36]. In one study [39], players exceeded protein recommendations while not consuming enough CHO.

Daily football training leads to musculoskeletal and tendinous tissue damage. Due to the important role of protein in muscle development and maintenance, football players should consume higher quantities of protein than the general population. According to the “food-first” philosophy, it is easy to achieve the recommended level of protein intake (1.6–2.2 g/kg BM/day) with a mixed diet. According to Burke et al. [54], the food-first philosophy states that nutrient delivery should come from whole foods and drinks, and there are situations wherein a “food-only” approach may not always be optimal for athletes. In special situations (e.g., dietary energy restriction, rehabilitation after injury), athletes require a higher proportion of protein in the diet [55,56,57]. To achieve the recommended amounts of nutrients in the diet of an athlete, and due to the difficulty in consuming large meals immediately after exercise, periodic supplementation with supplements, especially from group A according to the Australian Institute of Sport (AIS), may be considered. According to recent reports, food-first but not always food-only [58] football players in special cases should develop a personalized nutrition and supplementation plan via collaboration with their dietitian to optimize their performance.

### 4.4. Fat Intake in Football Players

Most of the studies included in this review indicated that the athletes studied consumed fat according to the recommended standard (20–35% of total energy) [21]. In contrast, in the study by Iglesias-Gutiérrez et al. [30], the athletes studied exceeded the recommended intake standard for this macronutrient (37 ± 5%). The study authors suggest that the high fat supply in football players’ diets may be dictated by high animal-protein intake [30]. The average proportion of fats in football players’ diets during the starting period was 31 ± 2%. A diet high in saturated fatty acids can negatively affect an athlete’s health [59]. Football players, when planning their individual diets, should pay attention to the ratio of total fat (<35% of total energy requirements), especially taking into account the optimal supply of saturated fatty acids (<10% of total energy requirements) [60].

### 4.5. Micronutrient Intake in Football Players

Due to the increased energy expenditure associated with intensive training and participation in matches, football players have a greater need for vitamins and minerals compared to the general population [42]. Regulatory ingredients play an important role in athlete nutrition by participating as precursors to metabolic pathways and physiological processes, which can help maximize adaptive return in athletes [61]. Studies included in the review showed the suboptimal supply of vitamin B_1_ and B_2_ [26], folic acid [24,40], vitamin A [24,26], vitamin D [24,40], vitamin C [26,40], calcium [24,26], magnesium [24,26], iron [24] and iodine [40]. In line with the food-first philosophy [54], nutrients (including vitamins and minerals) should come from standard foods and beverages, rather than from isolated ingredients in foods, dietary supplements or sports foods.

All vitamins and minerals are important for health and performance. However, according to the UEFA expert group statement [21], of particular note are vitamin D, iron and calcium. Vitamin D deficiency can lead to several health issues, including an increased risk of bone injuries, chronic musculoskeletal pain and viral respiratory tract infections [41,62,63]. Athletes with low 25-(OH)D levels (<30 ng/mL or <75 nmol/L) have also been shown to be at a higher risk of injury, including musculoskeletal injuries [64]. Close et al. [58] proposed recommendations for using dietary supplements in sport food-first but not always food-only. Authors have suggested several reasons why a food-only approach may not always be optimal for athletes, one of which concerns vitamin D [65].

Iron deficiency in the diet of athletes can have a negative impact on aerobic capacity [61,66], which may indirectly translate into the decreased effectiveness of training units and reduced adaptive capacity. Two studies included in this review showed an insufficient supply of this micronutrient in football players during the preparation period [24,26]. Football players, especially in-season, are at risk of iron deficiency [67,68]. Therefore, its serum concentration should be monitored, and dietary supply should be controlled. Proper doses of iron may be required to improve health through supplementation to correct any deficiency [58].

An optimal supply of calcium is necessary to maintain bone health, skeletal muscle function, cardiac contraction and nerve conduction. It has been shown that athletes can lose calcium with sweat [69]. Four papers included in the review showed an inadequate supply of calcium in the diets of the athletes studied [24,26,29,40]. To achieve the recommended dietary amount, football players should follow a mixed diet that includes calcium-rich foods (mainly dairy products, with smaller amounts from bony fish, legumes, certain nuts, plus fortified soy beverages and breakfast cereals). Consideration of the need for calcium supplementation may be required in cases of RED-S or in situations of increased calcium requirements (vegetarian/vegan diets) [58].

The reason for the non-adherence to dietary recommendations may be due to athletes’ knowledge on how to choose the right food for the main, pre- and post-workout meals. Physiological factors responsible for appetite can also contribute to an inadequate supply of energy, especially during long/high-intensity training periods [60]. Moreover, logistical issues, such as time constraints (short breaks between training sessions), can have an additional influence on insufficient nutrients intake.

### 4.6. Using Research Evidence in Practice

It is common knowledge that translating theoretical assumptions into practice is difficult to implement [70,71]. Translation of science depends on technical competency, personal attributes and practical skills (e.g., ability to build rapport with athletes, coaches, support staff). The delivery of nutritional knowledge should be supported by practical skills (e.g., ability to promote behavior change, cooking skills), resources and technological tools (e.g., relevant software, mobile applications) contained within the practitioner’s toolkit [72]. Therefore, the main goal of sports organizations/clubs should be hiring a qualified dietitian who should critically assess the translational potential of research and the applicability of these results in practice. Translating innovation in scientific research into practical applications for football players should be in the most accessible way, e.g., creating infographics, menu and recipe guides, video-based content, lectures or mobile applications. Nutritional education can be also be implemented by a special tool, such as the Athlete’s Plate (AP). The AP was developed to teach athletes how to design their plates depending on training load [73].

Furthermore, a necessary next step for knowledge translation are personal connections between sports organizations or clubs/staff members, and sports researchers/scientists may be a way to facilitate the use of nutrition research in sports. Developing a network of researchers to generate “credible knowledge” and facilitate the implementation of research in sports can help increase nutritional awareness among athletes [74]. The aforementioned activities can facilitate the implementation of research evidence in practice.

### 4.7. Limitations

This systematic review included only studies that assessed the intake of nutrients using validated methods. The underreporting of the quantitative estimates of dietary intake is one of the most common obstacles preventing the collection of accurate habitual dietary intake data [75,76]. In addition, data on dietary intake or anthropometric measurements were not provided by the authors of several studies included in this review. The missing data were calculated based on the available information in these studies, which may have reduced the accuracy of the results. It should not be underestimated that the delivery of and compliance with recommendations in the context of football is complex, because sports nutrition often has to deal with variable training loads, breaks and stops, injuries, return-to-play and complementary pharmacological management [77]. Moreover, the systematic review registration code is missing.

## 5. Conclusions

This review found that football players at a medium to high sporting level are not implementing the nutritional recommendations identified by the UEFA expert group. Among other things, insufficient supply of energy, CHO, vitamins and minerals (vitamin B_1_, B_2_, folic acid, vitamin A, C, D, E, calcium, magnesium, iron, iodine) were shown. Athletes do not periodize their CHO supply based on their training period. Moreover, the majority of athletes consume adequate protein and fat intake compared to recommendations.

In order to monitor athletes’ implementation of dietary recommendations and provide education on the subject, it is important for sports clubs to employ nutritionists. Nutritional periodization for macronutrient modification in the diet should take into account the nutritional goal, training period, position on the field and tactical tasks assigned to the player. This review highlights that further research should be focused on how to apply nutritional recommendations specific to athletes in specific positions on the field and be appropriate to the physical demands.

## Figures and Tables

**Table 1:** Population, intervention, comparison, outcome and study design (PICOS) criteria.

Parameter	Description
Population	Professional and semi-professional soccer/football players
Intervention/exposure	Baseline dietary intake
Comparison	Dietary intake compared to sports nutrition guidelines and recommendations
Outcome	Meeting/not meeting sports nutrition guidelines and recommendations
Study design	Cross-sectional, longitudinal and randomized controlled trials

**Table 2:** Search terms.

Concept	Key Words
Football (soccer) players	“football” OR “soccer” OR “players” OR “soccer players”
Dietary intake	“nutrient requirement” OR “nutritional supplement” OR “dietary supplement” OR “dietary intake” OR “daily food” OR “food intake” OR “dietary assessment” OR “dietary requirement” OR “sports nutrition” OR “food diary” OR “macronutrient” OR “nutrient needs” OR “dietary needs” OR “nutrient intake” OR “RDA”

**Table 3:** Eligibility criteria.

Inclusion	Exclusion
Original research (cross-sectional, observational, randomized controlled trials)	Reviews and other secondary research
English-language studies	Non-English-language studies
Studies published since 2011	Studies published up to 2011
Studies that include only male professional or semi-professional football/soccer players	Studies that include only non-professional or amateur football/soccer players, recreational only (non-competitive)
Studies that include only adult football/soccer players (i.e., >18 years of age)	Studies that include adolescent and child football/soccer players (i.e., <18 years of age)
Male football/soccer players	Female football/soccer players
Studies that include quantitative measures of dietary intake that can be converted into units of intake per day for each nutrient	Studies that include nutrition habits, attitudes, educational strategies, knowledge and those wherein dietary intake cannot be compared to other studies
Studies that assess dietary intake that uses a validated quantitative method of assessment (i.e., 7-day food diary, 7-day weighed-food diary, food records, 3-day food diaries, 24-h recall, weighed-food records, etc.) and therefore estimates absolute dietary intake	Studies that assess dietary intake using methods that provide dietary assessments of food groups, percentage of total energy, etc.
Published research	Unpublished research (i.e., theses)
Studies that include the dietary assessment of total energy, carbohydrate, protein, fat, micronutrient intake (i.e., iron in mg/day; calcium in mg/day, folate, etc.).	

**Table 4:** Participants’ characteristics in systematically reviewed studies.

Author	Country/League	Sports Level	*n*	Age (Years)	Height (cm)	Body Mass (kg)	Body Fat (%)	Fat Mass (kg)	Lean Mass (kg)
PRE-SEASON									
Noda et al., 2009 [26]	Japan	SP	31	19 ± 1	172 ± 6	66 ± 6			
Conejos et al., 2011 [40]	Valencia Football Club, Spain	P	22	22 ± 1					
Devlin et al., 2017 [32]	A-league, Australia	P	18			69 ± 7	14.7 ± 3.0	10.1 ± 2.5	
Devlin et al., 2017 [31]	A-league, Australia	P	18	27 ± 5	180 ± 7	76 ± 6	12.8 ± 1.9	8.7 ± 1.4	
Raizel et al., 2017 [24]	Brazil	P	19	20.7 ± 2	175 ± 9	72 ± 8	4.9 ± 1.5		
Książek et al., 2020 [29]	Ekstraklasa, Poland	P	26	27 ± 4	190 ± 10	78 ± 7	19.5 ± 3.3	15.6 ± 3.1	64.3 ± 6.4
Lee et al., 2020 [28]	Korea	SP	15	19.0 (19.0–19.5)	176 ± 5	70 ± 6	13.6 ± 2.6	9.5 ± 2.3	60.1 ± 4.5
Lee et al., 2021 [27]	Korea	SP	10	19 ± 1	176 ± 6	70 ± 6	13.3 ± 2.4	9.4 ± 2.4	60.4 ± 4.3
IN-SEASON									
Iglesias-Gutiérrez et al., 2012 [30]	U-21 First Division Soccer League Club, Spain	P	87	18 ± 2	179 ± 6	73 ± 7	10.5 ± 1.4		
Ono et al., 2012 [25]	Premier League, England	P	24						
Andrews and Itsiopoulos 2016 [36]	1 A-League, Australia	P	15	22 (18–37)					
	Victorian National Premier League, Australia	SP	31	21 (18–33)					
Bettonviel et al., 2016 [35]	Eredivisie, Netherlands	P	14	23 ± 4	181 ± 8	77 ± 9			
Anderson et al., 2017 [38]	Premier League, England	P	6	27 ± 3	180 ± 7	81 ± 9	11.9 ± 1.2	9.2 ± 1.6	65.0 ± 6.7
Anderson et al., 2017 [37]	Premier League, England	P	6	27 ± 3	180 ± 7	81 ± 9	11.9 ± 1.2	9.2 ± 1.6	65.0 ± 6.7
Devlin et al., 2017 [32]	A-league, Australia 1st point: start of season 2nd point: mid of season 3rd point: end of season	P	18			1st 69 ± 6	12.8 ± 1.9	8.7 ± 1.4	
						2nd 69 ± 6	12.6 ± 1.9	8.5 ± 1.4	
						3rd 70 ± 7	13.8 ± 2	9.5 ± 1.6	
Bonnici et al., 2018 [33]	BOV Premier League, Malta	SP	22	27 ± 4	175 ± 7	76 ± 8			
Anderson et al., 2019 [39]	Premier League, England	P	1	27	191	86	11.9	9.8	69.5
Brinksman et al., 2019 [34]	Eredivisie, Netherlands	P	41	23 ± 4	182 ± 6	78 ± 8	11.6 ± 2.4		68.6 ± 7.2

P—professional, SP—semi-professional.

**Table 5:** Reported energy and macronutrient intake of football players in the pre-season.

Author	Team Population (*n*)	Energy Intake (kcal)	Energy Intake (kcal/kg BM)	Proteins		Carbohydrate		Lipids		
				g	g/kg BM	g	g/kg BM	g	g/kg BM	% Energy
Noda et al., 2009 [26]	Collegiate football (soccer) players volunteered from the university (*n* = 31)	3006 ± 1052	45.6 ± 16	83 ± 31	1.3 ± 0.4	452 ± 162	6.9 ± 2.4	89 ± 36	1.3 ± 0.5	26 ± 4
Conejos et al., 2011 [40]	Men football (soccer) players during 7 days of training camp (*n* = 22)	3241 ± 1011	44.3 ± 6.4	143 ± 51	2.0 ± 0.7	371 ± 87	5.1 ± 1.2	123 ± 53	1.7 ± 0.7	34
Devlin et al., 2017 [31]	Football (soccer) players from one A-league soccer club (Australian) (*n* = 18)	2246 ± 549	29.7 ± 7.3	140 ± 35	1.9 ± 0.5	220 ± 76	2.9 ± 1.1	83 ± 31	1.1 ± 0.4	33 ± 9
Devlin et al., 2017 [32]	Football (soccer) players from one A-league soccer club (Australian) (*n* = 18)	2199 ± 550	32.1 ± 8	137 ± 40	1.9 ± 0.6	210 ± 76	2.9 ± 1.3	86 ± 35	1.1 ± 0.5	34 ± 12
Raizel et al., 2017 [24]	Football (soccer) players during the preparation phase for the Mato Grosso Governor Cup, an annual championship that qualifies for the Brazil Cup (*n* = 19)	2924 ± 920	40.7 ± 13	137	1.9 ± 0.8	392	5.4 ± 1.9	91.2	1.3 ± 0.5	28
Lee et al., 2020 [28]	Male Korean collegiate football (soccer) players recruited from a local university team competing in a national university league (*n* = 15)	3456 ± 435	49.6 ± 6.2							
Książek et al., 2020 [29]	Professional football (soccer) player recruited from one club competing in Polish league Ekstraklasa (*n* = 26)	2480 ± 389 Diet Excluding Supplements	31.5 ± 6.3	113 ± 18	1.4 ± 0.3	364 ± 70	4.6 ± 1.0	71 ± 12	0.9 ± 0.2	25 ± 3
		2656 ± 4 Diet Including Supplements	33.8 ± 7.2	119 ± 21	1.5 ± 0.3	398 ± 82	5.1 ± 1.2	74 ± 13	0.9 ± 0.2	24 ± 3
Lee et al., 2021 [27]	Male Korean collegiate football (soccer) players recruited from a local university team competing in a national university league (*n* = 10)	3342 ± 522	48.5 ± 7.5							

BM—body mass.

**Table 6:** Reported energy, macronutrient intakes of football players in in-season.

Author	Team Population (*n*)	Energy Intake (kcal)		Energy Intake (kcal/kg BM)	Proteins		Carbohydrate		Lipids		
					g	g/kg BM	g	g/kg BM	g	g/kg BM	%
											Energy
Iglesias-Gutiérrez et al., 2012 [30]	Male football (soccer) players from the Spanish First Division Soccer League Club, in the first half of the season, (*n* = 87)	2794 ± 526		38.5 ± 8.5	119 ± 24	1.6 ± 0.4	338 ± 70	4.7 ± 1.1	116 ± 30	1.6 ± 0.4	37 ± 5
Ono et al., 2012 [25]	Professional football (soccer) players were recruited from four Premier League clubs and a League One club (*n* = 24)	2648 to 4606			142 ± 23		505 ± 120				
Andrews and Itsiopoulos 2016 [36]	Professional football (soccer) players from 1 A-League club (*n* = 15)	2753 ± 475		34 ± 5	152 ± 28	1.9 ± 0.3	302 ± 72	3.5 ± 0.8	96 ± 32		30 ± 7
	Semiprofessional football (soccer) players from 4 National Premier League clubs (*n* = 31)	2587 ± 918		34.7 ± 10.7	149 ± 47	2.0 ± 0.6	290 ± 149	3.9 ± 1.8	86 ± 38		30 ± 7
Bettonviel et al., 2016 [35]	Senior professional football (soccer) players from the Dutch premier division (*n* = 14)	2988 ± 583		38.8 ± 7.6	145 ± 24	1.9 ± 0.3	365 ± 76	4.7 ± 0.7	97 ± 26	1.3 ± 0.3	29 ± 4
Anderson et al., 2017 [38]	Professional football (soccer) players of the English Premier League (*n* = 6) 1st author 2nd author 3rd independent researcher	1st 3174			208		347		106		
		2nd 3044			201		353		92		
		3rd 3013			194		332		101		
Anderson et al., 2017 [37]	Professional football (soccer) players of the English Premier League (*n* = 6) 1st point: Match day 2nd point: Training day	1st 2956 ± 374		36.7 ± 4.6			330 ± 98	6.4 ± 2.2			
		2nd 3789 ± 532		47.7 ± 6.6			508 ± 152	4.2 ± 1.4			
Devlin et al., 2017 [32]	Football (soccer) players from one A-league soccer club (Australian)(*n* = 18) 1st point: start of season 2nd point: mid of season 3rd point: end of season	1st 2247 ± 550		32.5 ± 7.9	140 ± 35	1.9 ± 0.5	220 ± 76	2.9 ± 1.1	83 ± 31	1.1 ± 0.4	33 ± 9
		2nd 2294 ± 550		33.2 ± 7.9	149 ± 40	2.0 ± 0.5	222 ± 87	2.9 ± 1.1	84 ± 34	1.1 ± 0.5	32 ± 11
		3rd 2318 ± 502		33.3 ± 7.2	157 ± 51	2.1 ± 0.7	213 ± 107	2.8 ± 1.4	86 ± 31	1.2 ± 0.5	33 ± 11
Bonnici et al., 2018 [33]	Semi-professional male football (soccer) players playing in the Malta BOV Premier League (*n* = 22)	2164 ± 498		28.6 ± 6.6	113 ± 30	1.5 ± 0.4	280 ± 76	3.7 ± 1.0	72 ± 12	1.0 ± 0.1	30 ± 5
Anderson et al., 2019 [39]	Professional goalkeeper of the English Premier League (*n* = 1)	3160 ± 381		36.9 ± 4.5	207 ± 36	2.4 ± 0.4	222 ± 54	2.6 ± 0.6	161 ± 26	1.9 ± 0.4	
Brinkmans et al., 2019 [34]	Senior professional football (soccer) players playing in the Dutch Eredivisie from different clubs (*n* = 41)	Training (*n* = 41)	2637 ± 823	34 ± 10.6	133 ± 43	1.7 ± 0.6	296 ± 104	3.9 ± 1.5	95 ± 41	1.2 ± 0.6	31 ± 6
		Match (*n* = 37)	3114 ± 978	40.1 ± 12.6	139 ± 46	1.8 ± 0.6	393 ± 137	5.1 ± 1.7	99 ± 41	1.3 ± 0.6	28 ± 7
		Rest (*n* = 39)	2510 ± 740	32.3 ± 9.5	116 ± 33	1.5 ± 0.5	289 ± 113	3.7 ± 1.4	94 ± 40	1.2 ± 0.5	32 ± 9
		Total group (*n* = 41)	2658 ± 693	34.3 ± 8.9	129 ± 36	1.7 ± 0.5	306 ± 86	4.0 ± 1.2	94 ± 33	1.2 ± 0.5	31 ± 5

BM—body mass.

**Table 7:** Reported energy and macronutrient intakes of football players in the pre-season, depending on their position on the field.

Author	Nutrients	Goalkeepers	Center Defender—Sides	Center Defender—Fullbacks	Defenders	Midfielders	Forwards	Total
Conejos et al., 2011 [40]	Energy							
	Kcal	2913.9 ± 1098.7			3537 ± 621	3346 ± 1482	3035 ± 693	3241 ± 1011
	kcal/kg BM	19.2 ± 7.5			50.9 ± 3.8	47.8 ± 7.7	46.3 ± 6.3	44.3 ± 6.4
	Protein							
	G	142.8 ± 100.1			145 ± 20	145 ± 57	139 ± 28	143 ± 51
	g/kg BM							
	Carbohydrate							
	G	320.3 ± 11.9			419 ± 98	382 ± 187	343 ± 93	371 ± 87
	g/kg BM							
	Fat							
	G	109.8 ± 45.3			125 ± 36	132 ± 63	120 ± 60	123 ± 53
	g/kg BM							
	% energy	35			32	36	35	34
Raizel et al., 2017 [24]	Energy							
	Kcal	3347 ± 609	2398 ± 409	2921 ± 974		2989 ± 1138	2746.2 ± 639.2	2924 ± 920
	kcal/kg BM	39.6 ± 7.2	34.0 ± 5.8	38.5 ± 12.9		44.3 ± 16.8	39.4 ± 9.2	40.7 ± 12
	Protein							
	G	129 ± 58	104 ± 35	131 ± 34		164 ± 65	101 ± 16	137 ± 54
	g/kg BM	1.8 ± 0.7	1.5 ± 0.5	1.8 ± 0.5		2.3 ± 1.0	1.4 ± 0.2	1.9 ± 0.8
	Carbohydrate							
	G	474 ± 97	318 ± 26	416 ± 206		379 ± 147	380 ± 98	391 ± 134
	g/kg BM	5.6 ± 1.2	5.5 ± 2.7	4.5 ± 0.4		5.6 ± 2.2	5.5 ± 1.4	5.4 ± 1.9
	Fat							
	G	97 ± 1	83 ± 20	77 ± 31		96 ± 43	95 ± 37	91 ± 36
	g/kg BM	1.2 ± 0.1	1.2 ± 0.3	1.0 ± 0.4		1.4 ± 0.6	1.4 ± 0.5	1.3 ± 0.5
	% energy	26	31	24		29	31	28

BM—body mass.

**Table 8:** Reported energy and macronutrient intakes of football players in the in-season, depending on their position on the field.

Author	Nutrients	Goalkeepers	Center Defenders	Fullbacks	Wingers	Defenders	Center Midfildiers	Central Defending Midfielders	Central Attacking Midfielders	Midfielders	Forwards	Total
Iglesias-Gutiérrez et al., 2012 [30]	Energy											
	kcal	2600 ± 641	2771 ± 582	2766 ± 452	2881 ± 385		2855 ± 475				2779 ± 659	2794 ± 526
	kcal/kg BM	33.3 ± 8.3	38.4 ± 10	39.2 ± 8.4	40.9 ± 6.0		39.9 ± 8.7				37.6 ± 8.3	38.5 ± 8.5
	Protein											
	g	115 ± 29	120 ± 21	117 ± 24	118 ± 22		117 ± 21				115 ± 27	119 ± 24
	g/kg BM	1.5 ± 0.4	1.7 ± 0.4	1.7 ± 0.4	1.7 ± 0.4		1.6 ± 0.3				1.6 ± 0.3	1.6 ± 0.4
	Carbohydrate											
	g	304 ± 35	313 ± 65	346 ± 58	352 ± 54		352 ± 72				352 ± 54	338 ± 70
	g/kg BM	3.9 ± 1.0	4.3 ± 1.1	4.9 ± 1	4.9 ± 0.8		4.9 ± 1.3				4.9 ± 0.8	4.7 ± 1.1
	Fat											
	g	111 ± 32	124 ± 34	110 ± 28	121 ± 21		118 ± 32				121 ± 21	116 ± 30
	g/kg BM											
	% energy	38 ± 4	40 ± 4	36 ± 5	38 ± 3		37 ± 6				38 ± 3	37 ± 5
Anderson et al., 2017 [38]	Energy											
	kcal		2961	2905	3563			3166	3701		2817	3186 ± 367
	kcal/kg BM		33.3	39.6	50.2			39.8	47.2		31.4	39.7 ± 4.6
Anderson et al., 2019 [39]	Energy											
	kcal	3160 ± 381										
	kcal/kg BM	36.9 ± 4.5										
	Protein											
	g	207 ± 36										
	g/kg BM	2.4 ± 0.4										
	Carbohydrate											
	g	222 ± 54										
	g/kg BM	2.6 ± 0.6										
	Fat											
	g	161 ± 26										
	g/kg BM	1.9 ± 0.4										
	% energy											
Brinksman et al., 2019 [34]	Energy											
	kcal	2606 ± 586				2864 ± 699				2534 ± 550	2602 ± 874	2658 ± 693
	kcal/kg BM	29.1 ± 6.5				36.2 ± 8.8				35.3 ± 7.7	33.2 ± 11.1	34.3 ± 8.9
	Protein											
	g	134 ± 45				136 ± 45				125 ± 33	126 ± 29	129 ± 36
	g/kg BM	1.5 ± 0.5				1.7 ± 0.5				1.8 ± 0.5	1.6 ± 0.4	1.7 ± 0.5
	Carbohydrate											
	g	322 ± 64				328 ± 90				286 ± 53	301 ± 116	306 ± 86
	g/kg BM	3.6 ± 0.8				4.2 ± 1.4				4 ± 0.8	3.8 ± 1.4	4 ± 1.2
	Fat											
	g	80 ± 19				104 ± 30				92 ± 34	93 ± 39	94 ± 33
	g/kg BM	0.9 ± 0.2				1.3 ± 0.4				1.3 ± 0.5	1.2 ± 0.5	1.2 ± 0.5
	% energy	27 ± 2				32 ± 4				31 ± 6	31 ± 5	31 ± 5

BM—body mass.

## Data Availability

Not applicable.
